# Unimodal and cross-modal prediction is enhanced in musicians

**DOI:** 10.1038/srep25225

**Published:** 2016-05-04

**Authors:** Eliana Vassena, Katty Kochman, Julie Latomme, Tom Verguts

**Affiliations:** 1 Department of Experimental Psychology, Ghent University, Belgium; 2 Institute for Psychoacoustics and Electronic Music, Ghent University, Belgium

## Abstract

Musical training involves exposure to complex auditory and visual stimuli, memorization of elaborate sequences, and extensive motor rehearsal. It has been hypothesized that such multifaceted training may be associated with differences in basic cognitive functions, such as prediction, potentially translating to a facilitation in expert musicians. Moreover, such differences might generalize to non-auditory stimuli. This study was designed to test both hypotheses. We implemented a cross-modal attentional cueing task with auditory and visual stimuli, where a target was preceded by compatible or incompatible cues in mainly compatible (80% compatible, predictable) or random blocks (50% compatible, unpredictable). This allowed for the testing of prediction skills in musicians and controls. Musicians showed increased sensitivity to the statistical structure of the block, expressed as advantage for compatible trials (disadvantage for incompatible trials), but only in the mainly compatible (predictable) blocks. Controls did not show this pattern. The effect held within modalities (auditory, visual), across modalities, and when controlling for short-term memory capacity. These results reveal a striking enhancement in cross-modal prediction in musicians in a very basic cognitive task.

Music is a universal attribute to all human cultures and pervasive in daily life^1^,^2^,^3^. Advanced musical practice involves skills in processing various kinds of stimuli. First, musicians are highly trained in the memorization of auditory stimuli, from simple tones to complex rhythms and harmonic structures^4^. Second, musicians read symbolic visual stimuli associated with those tones. Third, musicians produce tones by performing automatized, refined actions involving haptic feedback in coordination with their environment^5^,^6^. These components are combined in a rapidly evolving processing stream, and yet organized in a meaningful sequence, which produces the pleasant stimulus the listener perceives as music^7^,^8^.

The complexity of musical training suggests that consistent exposure and expertise may be associated with measurable effects in several cognitive functions^9^, as well as on brain plasticity^10^. Musicians show enhanced auditory-perception skills, such as pitch discrimination, temporal order judgment^11^ and discrimination of psychoacoustic features^12^. Moreover, structural and functional changes in brain regions dedicated to auditory processing have been consistently reported^13^. These auditory-perceptual advantages suggest that musical expertise is associated with improved temporal discrimination and attentional capacity^14^.

The generalization of these benefits to other cognitive functions remains debated^15^. A popular study reported that exposure to a 10-minute fragment by Mozart improved spatial reasoning^16^. This result had great media resonance and was named “Mozart effect” by the press, conveying the idea that classical music could in fact improve cognitive skills. Although not consistently replicated^17^, this result stimulated further research testing whether musical training provides benefits beyond auditory perception, yielding controversial results.

On the one hand, several studies have reported advantages for musicians in diverse cognitive domains. Musicians have shown better reproduction of time intervals for both auditory and visual intervals^18^, as well as better reproduction of multimodal sequences^12^. Musicians have also performed better in judging whether auditory and visual information was presented synchronously or asynchronously in musical videoclips^19^. Musicians also outperform controls in attention and visuo-spatial tasks such as detecting single elements in complex objects, detecting letters among digits^20^, and line bisection^21^. Finally, children exposed to 9-months of musical training have shown increased reading abilities as well as pitch discrimination in speech^22^. These findings suggest a cross-modal transfer of benefits for musicians beyond the musical domain and beyond the auditory modality.

On the other hand, several studies have found selective benefits to musical and auditory processing, with no generalization to other modalities. For example, advantages in attentional performance were reported only for auditory, but not for visual attention tasks^12^,^23^. Also, musicians proved selectively better at reproducing auditory sequences but not audio-visual sequences^24^. Additionally, no advantage was reported in learning sequence structure after passive listening. Lastly, musicians showed improved detection of audio-visual asynchrony but only with music and not with speech^25^. However, an important caveat is that most of these studies present correlational evidence, showing better performance in musicians compared to controls. Although informative, one cannot infer a causal influence of musical training on cognitive skills (be it selective to the auditory and musical domain or more general). Causal evidence remains sparse in the literature and should receive further attention in future research.

This overview suggests auditory processing benefits, possibly deriving from the extensive expertise that musicians acquire in fast-scale temporal processing. However, the evidence for advantages beyond the auditory domain remains varied.

A potential benefit on the core cognitive process of prediction has been hypothesized, but not directly tested. Prominent cognitive theories such as predictive coding and reinforcement learning suggest that cognitive processing proceeds by prediction^26^,^27^,^28^,^29^,^30^. In this framework, each stimulus or sequence leads to a prediction of the upcoming stimulus. Predicted and actual stimulus are compared, and this discrepancy is termed prediction error. Prediction errors drive both cognitive processing and learning^31^. Perception of musical rhythm and meter have been framed in the context of predictive theories^32^, as well as the relationship between perception and action in musical performance^33^. Error (and prediction error) minimization is the core concept shared by these accounts. Furthermore, it has been proposed that the surprise associated with prediction error carries an affective component, and an ideal amount of surprise (not too much but not too little) drives affective reaction to music, as well as guiding expert musicians in pleasing their audience^34^. From the empirical point of view, studies reported an advantage for musicians in detecting auditory prediction errors, both with simple sounds and complex harmonic structures^35^,^36^. The neural signature of deviance detection also reflects this facilitation. Rhythmic deviance also elicits error-related neural activity^37^. Moreover, expert musicians have shown neural correlates of error detection even before performing and incorrect action, supposedly arising from a continuous fast monitoring of predictions and outcomes^38^.

Taken together, these findings support a pivotal role of prediction in music perception and performance. An further intriguing possibility is that musical expertise might be associated with improved prediction skills. The goal of the current study was to test this hypothesis with a standard cognitive task involving basic stimulus-outcome prediction skills, outside the musical domain. Given that prediction applies to any stimulus sequence irrespective of its modalities, we hypothesized that this facilitation may extend to non-auditory (e.g. visual) and even cross-modal sequences (i.e., when an auditory stimulus is predictive of a visual one and vice-versa). The advantage should manifest as increased sensitivity to prediction errors, as a consequence of increased encoding of the statistical structure of the environment in both unimodal and cross-modal conditions. To test this, we implemented a cross-modal cueing paradigm with auditory and visual stimuli. We implemented different levels of predictability and thus prediction errors by different frequencies of compatible and incompatible cue-target pairs. Moreover, we administered two control tasks to measure verbal and visuo-spatial short-term memory. The goal was to determine whether the hypothesized difference would be specific to prediction, or simply accountable to differences in short-term memory capacity.

## Results

In the cross-modal cueing task, overall accuracy was 78% ± 0.4. Accuracy rates were averaged for each subject and for each condition, and subjected to a rANOVA. No significant effect of group was found (*F*_(1,27)_ = 0.524, *p* = 0.48), showing that accuracy did not differ between musicians and controls. A significant main effect of compatibility was observed (*F*_(1,28)_ = 4.57, *p* < 0.05, *η*^2^ = 0.14), with higher accuracy for compatible trials (M = 0.96 ± 0.02) relative to incompatible trials (M = 0.95 ± 0.02).

RTs were averaged for each subject and for each condition. Error trials (4.7%) were excluded from further analysis. To minimize the impact of outliers, trials with RTs higher or lower than 2.5 standard deviations of the individual mean were also excluded (2.9%). Trimming means by removing outlying observations is a common way of making the mean a more robust measure of central tendency^39^, and a 2.5 standard deviations cut-off is a commonly used convention in the field^40^. Subsequently, we tested the assumption of normality of the residuals with the Shapiro-Wilk test. All p-values were larger than 0.05, confirming that the residuals were normally distributed. For 2 out of the 16 conditions the p-values were still rather small (0.08 and 0.06). To test the robustness of our results, we log-transformed the data and run the main analysis again. All significant main effects and interactions reported in the main analysis were preserved when tested on the log-transformed data.

**Figure 1:**
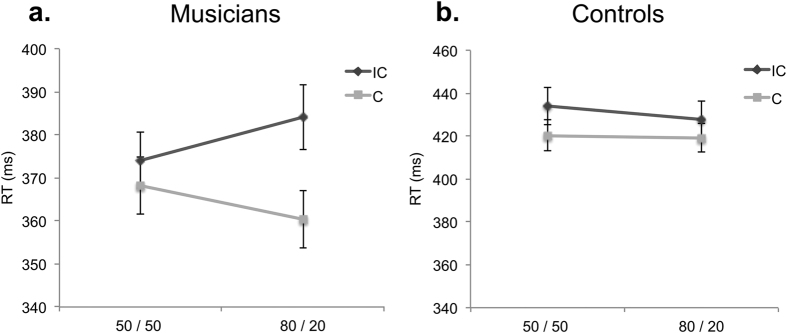
Group × compatibility frequency × compatibility interaction. (**a**) Average RTs for musicians in compatible (C) and incompatible (IC) trials, as a function of compatibility frequency in the block (50/50, 80/20). The error bars represent one standard error of the mean. (**b**) Average RTs for controls in compatible (C) and incompatible (IC) trials, as a function of compatibility frequency in the block (50/50, 80/20). The error bars represent one standard error of the mean.

Crucially, the rANOVA revealed a significant interaction group × compatibility frequency × compatibility (*F*_(1,28)_ = 6.24, *p* < 0.05, *η*^2^ = 0.18, see Fig. 1), with musicians showing a stronger influence of compatibility frequency (enhanced compatibility effect in the 80/20 condition) as compared to controls.

Pairwise comparisons revealed a significant difference for musicians between compatible and incompatible trials in the 80/20 condition (*t*_(14)_ = −20.17, *p* = 0.001), but not in the 50/50 condition (*t*_(14)_ = −4.9, *p* = 0.21). Thus, musicians were relatively disadvantaged for incompatible targets, but only in the 80/20 condition. Conversely, controls showed no difference between compatible and incompatible trials in the 80/20 condition (*t*_(14)_ = −1.68, *p* = 0.12) but did show a small difference between compatible and incompatible trials in the 50/50 conditions (*t*_(14)_ = −2.61, *p* = 0.02). However, only the difference for the musicians between compatible and incompatible trials in the 80/20 condition remained significant after Bonferroni correction for multiple comparisons. This interaction shows increased sensitivity in musicians to compatibility frequency, suggesting a better representation of the statistical structure of the block. This translated in an increased compatibility effect, when incompatible trials were less frequent. Crucially, the cue modality (*F*_(1,28)_ = 0.01, *p* = 0.93) or target modality (*F*_(1,28)_ = 0.73, *p* = 0.4) did not interact with the 3-way group × compatibility frequency × compatibility interaction, indicating that the effect holds across cue and target modalities.

Furthermore, a main effect of group was observed (*F*_(1,28)_ = 10.49, *p* < 0.01, *η*^2^ = 0.27), with musicians showing overall faster RTs than controls. Pairwise comparisons across compatibility frequency and compatibility frequency conditions revealed that musicians reacted faster than controls in all conditions: to compatible targets and incompatible targets in random blocks (C *t*_(28)_ = −3.39, p = 0.002, IC *t*_(28)_ = −3.46, *p* = 0.002), and to compatible and incompatible targets in mainly compatible blocks (C *t*_(28)_ = −3.6, p = 0.001, IC *t*_(28)_ = −2.37, *p* = 0.025). This last comparison however, was not significant after applying a Bonferroni correction for multiple comparisons.

Additionally, there was a main effect of target modality (*F*_(1,28)_ = 189.51, *p* < 0.001, *η*^2^ = 0.87), with faster RTs to visual targets, and a main effect of compatibility (*F*_(1,28)_ = 16.61, *p* < 0.001, *η*^2^ = 0.37), with faster RTs in compatible trial. A significant group × target modality interaction was also observed (*F*_(1,28)_ = 5.6, *p* < 0.05, *η*^2^ = 0.17): Musicians responded faster to visual targets compared to auditory targets (*t*_(14)_ = −7.81, *p* < 0.001, mean difference −87.6 ms); controls also responded faster to visual targets (*t*_(14)_ = −11.8, *p* < 0.001, mean difference −123.9 ms); however, the difference for controls was larger, thus driving the interaction. This interaction might reflect a facilitation for musicians in responding to auditory stimuli. Furthermore, there was a significant cue modality × target modality interaction (*F*_(1,28)_ = 32.93, *p* < 0.001, *η*^2^ = 0.54), with faster RTs to visual as compared to auditory targets (*t*_(29)_ = −12.79, *p* < 0.001), but no significant difference between visual and auditory cues (*t*_(29)_ = 0.21, *p* < 0.84). A target modality × compatibility frequency interaction was also reported (*F*_(1,28)_ = 5.29, *p* < 0.05, *η*^2^ = 0.16): RTs were faster for visual compared to auditory targets in both the 80/20 condition (*t*_(29)_ = −12.38, *p* < 0.001, mean difference = −101.37 ms) and 50/50 condition (*t*_(29)_ = −12.56, *p* < 0.001, mean difference = −110.18 ms), with a larger difference for the latter.

Subsequently, performance on the short-term memory tasks was analyzed. The overall verbal short-term memory capacity score was 74.03 ± 6.01. No significant differences were observed between musicians (M = 76.2 ± 7.54) and controls (M = 71.87 ± 9.6, *t*_(28)_ = 0.34, *p* = 0.73). The overall visuo-spatial short-term memory capacity score was 74.4 ± 4.36. No significant differences were reported between musicians (M = 79.8 ± 7.01) and controls (M = 74.4 ± 4.36, *t*_(28)_ = 1.28, *p* = 0.22).

Although there was no group effect in short-term memory, in order to further ensure that the RT effects in the cross-modal cueing task could not be explained by differences in short-term memory capacity, the main rANOVA on RTs was repeated, including both short-term memory scores as covariates. No significant interaction of any factor with short-term memory scores was observed. Moreover, the most relevant group × compatibility frequency × compatibility was preserved (*F*_(1,28)_ = 7.07, *p* < 0.05, *η*^2^ = 0.21), showing that the core finding (Fig. 1) is not driven by differences in short-term memory capacity.

## Discussion

This study investigated the basic cognitive skill of prediction in musicians and non-musicians. We hypothesized an advantage for musicians in encoding predictable event sequences. The results can be summarized as follows. First, musicians showed enhanced prediction relative to controls, expressed as increased sensitivity to statistical block structure (compatibility frequency) in a very basic cueing task. Second, modulation by musical expertise held across modalities, revealing a striking cross-modal generalization. This shows increased prediction skills in musicians as compared to controls irrespective of event modality. Third, enhanced prediction could not be explained by short-term memory differences.

Earlier work addressed the role of prediction in music and auditory processing. One conclusion was that regular sound sequences generate predictions and prediction errors at several hierarchical levels, which can determine the pleasurableness of the sequence^41^. Showing better prediction skills in musicians in a very basic cueing task indicates that the prediction machinery used in musical processing is rooted in basic cognitive prediction mechanisms.

Additionally, we reported an overall advantage for musicians, who responded faster in all conditions and irrespective of modality (although no differences in accuracy at any of the tasks were found). Moreover, the group × target modality interaction, suggested to some extent faster processing of auditory targets. On the one hand, this results is compatible with prominent accounts stating that musical training results in fine-tuning and increased efficiency and precision of the auditory system^13^, also generalizing to speech^42^,^43^. On the other hand, we report an advantage in prediction for musicians irrespective of cue and target modality, thus advocating for a non-selective effect.

In conclusion, this study provides several avenues for future work. First and foremost, we did not investigate causality. Our data is correlational in nature and does not allow for the drawing of inferences on the effect of musical training on cognition. In fact, a plausible alternative interpretation could be that people with better prediction skills become interested in music as a consequence of their natural abilities, and are presumably more suited to pursue music professionally in life. In order to disentangle the origin of such differences between musicians and non-musicians future studies should administer musical training to naive subjects and measure prediction before and after training. In addition to testing for causal relationship, this would allow investigating the amount of training required to induce measurable benefits. This might be particularly relevant in the context of longitudinal studies addressing the benefits of musical education.

Second, we did not distinguish between types of musical expertise or years of training, variables that were relevant in earlier research^44^. The extent of the advantage might depend on musical instrument, as well as vary as a function of years of training.

Third, our sample size was rather limited. Future studies should aim for a larger amount of participants in each group to increase power and generalizability of the results.

Finally, the role of prediction in cognitive processing has been widely studied in computational neuroscience (e.g., reinforcement learning^45^, predictive coding^26^,^31^,^46^). Here, prediction is central in perception, learning, memory, decision-making, and action selection. It is an exciting open question to what extent training prediction skills as implemented in musical practice, may improve such domain-general abilities.

## Methods

### Participants

Thirty subjects participated to the study (age range 17–33, M = 19.5, SD = 3.2), recruited among Ghent University students who earned credits for participation. The sample size was determined a priori, following earlier conventions in music research^11^,^18^,^21^. All participants provided written informed consent. Fifteen were selected for their musical expertise (nine males) with the following requirements: a minimum of five years of playing a musical instrument; having followed formal musical training in a music school; practicing the instrument on a daily basis; and currently playing the instrument at the time of the study. Fifteen control participants were selected (five males), where the listed requirements were exclusion criteria. Control participants and musicians did not differ in age (*t*_(28)_ = 0.11, *p* = 0.91) group. All participants gave written informed consent before participation. The experiment was conducted under the General Ethical Protocol for scientific research at the Department of Psychology and Educational Sciences of Ghent University, approved by the department’s ethical committee. The procedure was in accordance with the guidelines provided within such protocol.

### Procedure

After completing the informed consent, participants performed the main task (cross-modal cueing task), followed by two control tasks measuring verbal short-term memory (verbal span task) and visuo-spatial short-term memory (Corsi block tapping task). All tasks were programmed in E-prime 2.0 (Psychology Software Tools, Pittsburgh, PA) and presented on a 15″ computer screen. Headphones were used to present auditory stimuli.

### Cross-modal cueing task

**Figure 2:**
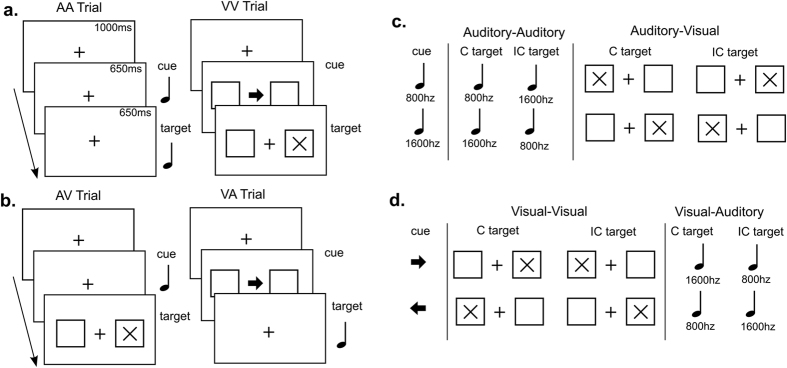
Task structure. (**a**) Task timing with an example of the two unimodal trial types: auditory-auditory (AA) with auditory cue and auditory target (tones); visual-visual (VV) with a visual cue (arrow) and visual target (X). (**b**) Example of the two cross-modal trial types: auditory-visual (AV) with auditory cue (tone) and visual target (X); visual-auditory (VA) with visual cue (arrow) and auditory target (tone). (**c**) Cue-target combinations and compatibility for AA and AV trials. From left to right: Auditory cues (low tone 800 hz, high tone 1600 hz); compatible (C) and incompatible (IC) auditory targets (AA trial); compatible (C) and incompatible (C) visual targets (AV trial). (**d**) Cue-target combinations and compatibility for VV and VA trials. From left to right: Visual cues (left or right pointing arrow); compatible (C) and incompatible (C) visual targets (VV trial); compatible (C) and incompatible (C) auditory targets (VA trial).

Each trial started with a fixation cross (see Fig. 2). A first stimulus was presented as a cue (650 ms). The cue was followed by a target (650 ms), to which the participants had to respond as quickly and as accurately as possible, with a maximum response time limit of 1650 ms. In all trials, pressing the response key terminated the trial. Cue and target could be compatible or incompatible. In auditory-auditory trials, cue and target were auditory stimuli. Each of them could be a low pitch tone (800 Hz) or high pitch tone (1600 Hz, 650 ms). At the target, participants responded by indicating if the tone was low (left key press) or high (right key press). In compatible trials, the target matched the pitch of the cue, while in incompatible trials it did not. In visual-visual trials, two squares were presented, one to the left and one to the right of the fixation cross. The cue consisted of an arrow pointing left or right appearing in the center of the screen. The target stimulus consisted of an X, appearing either in the left square or in the right square. Participants had to indicate if the target stimulus X was on the left (left key press) or on the right (right key press). In compatible trials, the X appeared in the same direction as the arrow pointed, while in incompatible trials it appeared on the opposite side. In auditory-visual trials, the auditory cue was followed by the visual target. Trials were considered compatible when a low tone was followed by an X on the left, and a high tone by an X on the right. Trials were considered incompatible when a low tone was followed by an X on the right and when a high tone was followed by an X on the left. In visual-auditory trials the visual cue was followed by the auditory target. Trials were considered compatible when an arrow pointing left was followed by a low tone and an arrow pointing right by a high tone. Trials were considered incompatible when a left-pointing arrow was followed by a high tone and a right-pointing arrow by a low tone.

Cue modality and target modality were manipulated across blocks, yielding AA, AV, VA and VV blocks. Each of these block types occurred with 80% compatible and 20% incompatible trials (80/20 condition, 40 trials per block, 8 of which incompatible), or with 50% compatible and 50% incompatible trials (50/50 condition, 28 trials per block). This represents the crucial manipulation, as the 80/20 condition is characterized by a statistical structure that prompts prediction. This resulted in 8 blocks, repeated 4 times each, for a total of 32 blocks and 1088 trials. The order of presentation of these blocks was randomized, as well as the order of presentation of trials within a block.

To summarize, the design implemented the following factors: cue modality (auditory or visual), target modality (auditory or visual), compatibility frequency in the block (80% or 50%), and compatibility in the trial (compatible or incompatible). The task lasted about 45 minutes.

### Verbal short-term memory task

To control for verbal short-term memory capacity, a letter span task was administered^47^. At the start of every trial, a blank screen was presented (1500 ms). A sequence of 4 consonants followed, each remaining on the screen for 1200 ms (inter-letter blank of 250 ms). After a 1500 ms retention period, participants were instructed to reproduce the sequence by pressing the corresponding keys on the keyboard in the correct order. The length of the string to be retained increased by one every time that the participant correctly reported three sequences of the current length. The maximum string length was 9. Failing to reproduce a sequence for three consecutive trials would terminate the task, constituting the span length for that participant. A first practice trial with feedback was presented in the beginning. The total task duration was 5 minutes on average.

### Visuo-spatial short-term memory task

To control for visuo-spatial short-term memory capacity, a Corsi block tapping task was administered to all participants^48^. At the start of every trial, a grid of 9 grey-colored squares (35 × 35 mm) was presented (1200 ms). Three of the squares would sequentially turn black (each for 1000 ms with 500 ms in between). After a 1000 ms blank screen, participants were asked to report the order of appearance of the squares, by clicking on the square in the order in which they were presented. Correctly reproducing the order in three trials would increase the number of squares by one. The maximum number of squares was 4. Failing to reproduce the order of presentation in three consecutive trials would terminate the task, providing the span length for that participant. A first practice trial with feedback was presented in the beginning of the task. The total task duration was on average 5 minutes.

### Analysis

First, accuracy at the cross-modal cueing task was analyzed. A repeated measures analysis of variance (rANOVA) was performed on the accuracy data, with between-subjects factor group (musicians, controls), and within-subjects factors cue modality (auditory, visual), target modality (auditory, visual), compatibility frequency in the block (80/20, 50/50), and compatibility in the trial (compatible, incompatible). Second, the reaction times (RTs) of the cross-modal cueing task were analyzed. A rANOVA was conducted on this data, with between-subjects factor group (musicians, controls), and within-subjects factors cue modality (auditory, visual), target modality (auditory, visual), compatibility frequency in the block (80/20, 50/50), and compatibility in the trial (compatible, incompatible.) Third, the scores at the short-term memory tasks were computed. Following conventions from the literature, in both tasks accuracy was calculated as the longest sequence correctly reproduced, multiplied by the total number of correctly reproduced sequences. For example, in the letter span tasks, a participant who correctly reproduced a 6-letter span and overall reproduced 11 spans correctly received an accuracy score of 66. Two-sample t-tests were performed on the verbal short-term memory task scores and visuo-spatial short-term memory task to test for differences in short-term memory capacity between musicians and controls.

## Additional Information

**How to cite this article**: Vassena, E. *et al*. Unimodal and cross-modal prediction is enhanced in musicians. *Sci. Rep*. **6**, 25225; doi: 10.1038/srep25225 (2016).
